# Basal cell skin cancer with metastasis to subcutaneous fat—a case report

**DOI:** 10.3389/fsurg.2025.1575461

**Published:** 2025-06-19

**Authors:** Veronika Rumyantseva, Anna Sukhotko, Victor Sukharev, Anna Bumbu, Evghenii Zakurdaev, Serghei Covantsev

**Affiliations:** ^1^ Department of Surgery, Russian Medical Academy of Postgraduate Education, Moscow, Russia; ^2^ Department of Oncology, S.P. Botkin Clinical Hospital, Moscow, Russia

**Keywords:** basal cell carcinoma, metastasis, axillary lymph node, subcutaneous fat, nonmelanoma skin cancers

## Abstract

Basal cell carcinoma (BCC) is a malignant neoplasm of the skin that originates from the cells of the basal layer of the epidermis and is the most common skin cancer worldwide, accounting for more than 75% of all nonmelanoma skin cancers. About 4 million BCCs are diagnosed annually worldwide. Despite being the most common skin tumor, metastasis is rare and occurs in 0.0028%–0.55% of cases. In this case report, we present a rare case of giant basal cell carcinoma with metastasis to the subcutaneous fat in a 69-year-old woman.

## Introduction

Basal cell carcinoma (BCC) is a malignant skin neoplasm that originates from the basal layer of the epidermis and is the most common skin cancer worldwide, accounting for more than 75% of all nonmelanoma skin cancers (NMSCs) (1). Approximately 4 million BCCs are diagnosed annually worldwide (2). Risk factors include ultraviolet light exposure, fair skin, radiation therapy, long-term arsenic exposure, and poor immune function (3).

Despite being the most common skin tumor, metastasis is rare, occurring in 0.0028%–0.55% of cases (4). The first case of BCC with metastasis was described by Beadles in 1894 and since then only 389 similar cases have been documented (4, 5). Metastases can be lymphatic or hematogenous, with the most common sites being regional lymph nodes, followed by lungs and bones (4).

In this report, we present a rare case of giant BCC of the skin in a 69-year-old woman with metastasis to the subcutaneous fat, initially considered as axillary lymph node.

## Clinical case

**Figure 1:**
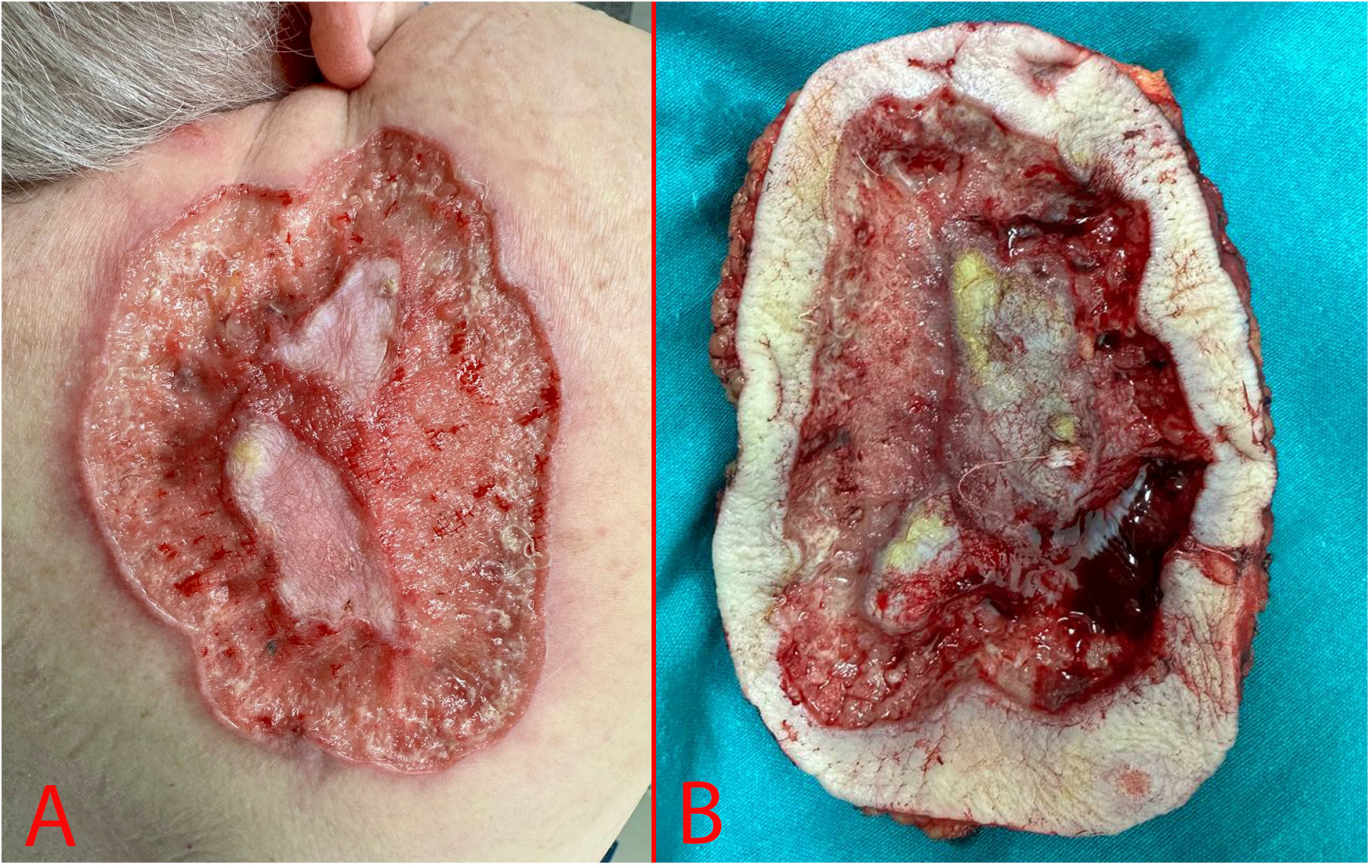
Macroscopic specimen. **(A)** – Preoperative image; **(B)** – Postoperative specimen.

**Figure 2:**
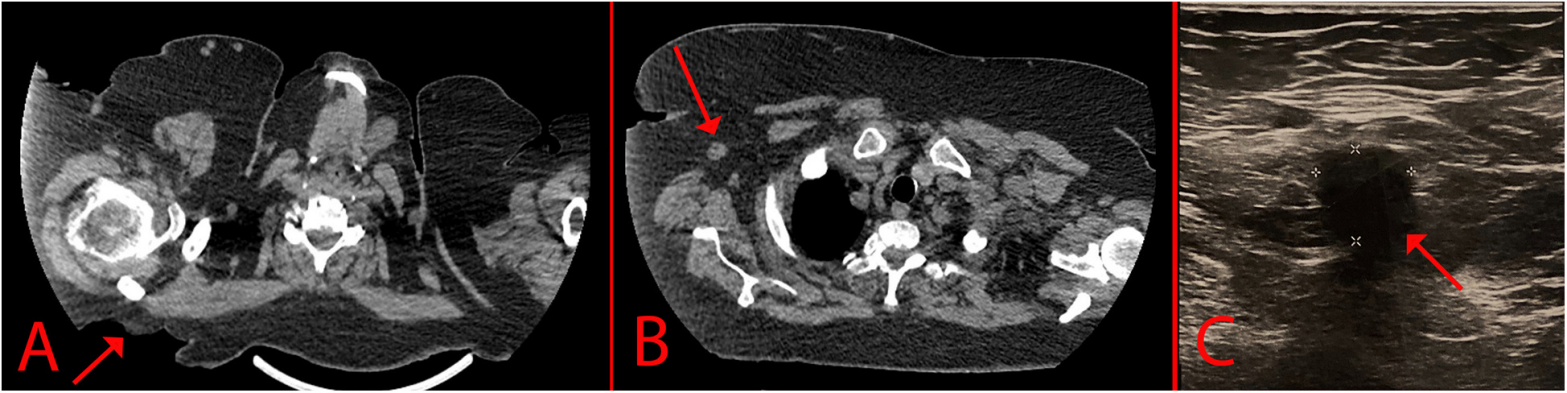
Imaging of the lesion. **(A)** – CT scan that indicates a skin defect; **(B)** – Enlarged axillary lymph node; **(C)** – USG of the axillary lymph node.

A 69-year-old woman, was hospitalized in the oncological surgical department with complaints of a skin lesion on her back. She considered herself ill for 12 years, when she first discovered an ulcer on the skin in the suprascapular region (Figure 1). During this period, she treated herself on her own with herbal ointments (could not specify which) and did not consult a doctor. Based on Fitzpatrick skin phototype classification she was type II. In May 2024, she was hospitalized on an emergency basis with an abscess of the right suprascapular region. She undergone a biopsy of the mass on 24.05.2024. According to the histological examination results, the histology most likely corresponds to skin BCC. After discharge, she was referred for consultation to the oncology center where the histology was reviewed. Based on the results of the histological examination the morphological picture was consistent with skin BCC. In order to verify the diagnosis, an immunohistochemical study was requested. According to the immunohistochemical study dated 20.06.2024, the morphological picture and the nature of the expression of markers (GATA binding protein 3: +, cytokeratin [CK] 5/6: -, Erythroblast transformation-specific Transcription Factor [ERG]: -, tumor protein P63: +) corresponded to BCC. Further examination included computer tomography (CT) of the chest, which demonstrated a defect in the skin and subcutaneous fat in the right suprascapular region, and a rounded contrast enhanced lymph nodes with altered internal architecture at the level of the right axillary fossa (Figure 2).

The patient also undergone USG for screening of regional and distant lymph nodes. In the right axillary region there was a single hypovascular lymph node measuring 12 × 11 mm with loss of corticomedullary differentiation (Figure 2C). Core-needle biopsy of the axillary lymph node was performed under USG-guidance. The biopsy contained fragments of partially sclerotic fibrofatty tissue with foci of necrosis with small scattered carcinoma complexes. In order to verify the metastasis, an immunohistochemical study was requested. According to the immunohistochemical study 29.07.2024, the morphology and immunophenotype (GATA3 +, CK 5/6: -, ERG: -, tumor protein P63: +) corresponded to a metastasis of BCC. An oncological consilium decided for surgical treatment as a first step.

The patient was admitted with a diagnosis of BCC of the skin of the right suprascapular region cT3N1M0, III stage. Concomitant diseases included hypertension, hyperlipidemia, type 2 diabetes mellitus, class 2 obesity, chronic kidney disease (stage 2), and cholelithiasis.

During examination we determined an area of skin ulceration measuring 18 × 15 cm in the right suprascapular region with uneven edges, a necrotic area in the center, and high bleeding upon contact (Figure 1).

Taking into account clinical, radiological and histological studies, a decision was made to perform surgical treatment in the amount of wide excision of the skin neoplasm with reconstruction and axillary lymphadenectomy.

**Figure 3:**
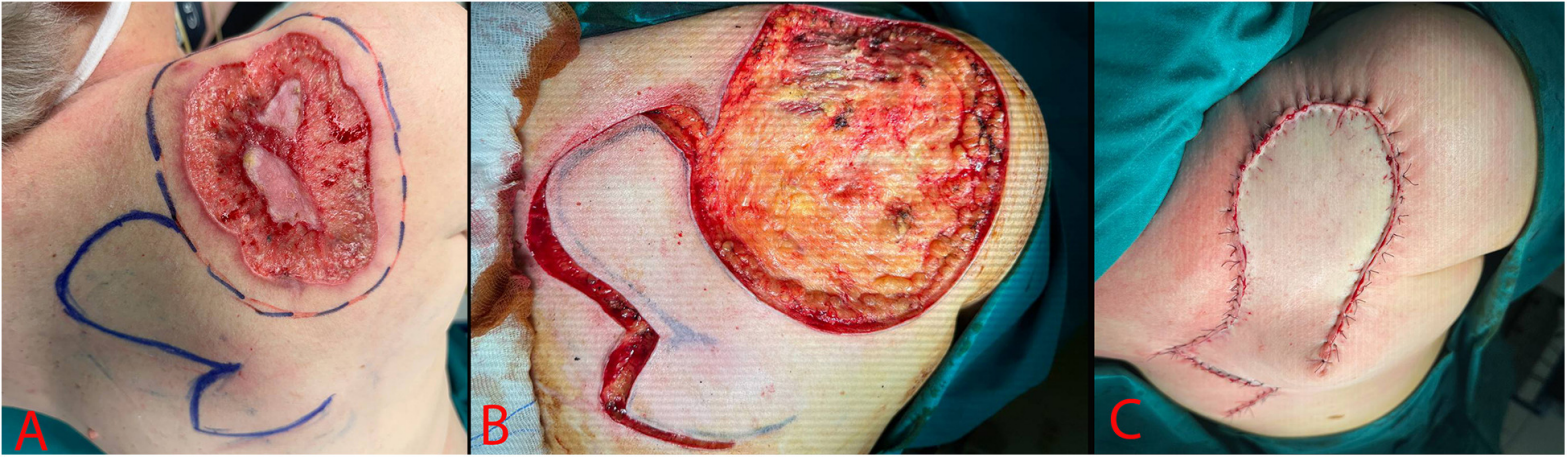
Surgery stages. **(A)** – Preoperative marking; **(B)** – Excised skin defect; **(C)** – Postoperative image.

The edges of the neoplasm excision and the rotating multi-lobe flap were marked with a permanent marker in the preoperative period. Under combined endotracheal anesthesia, the patient was transferred to the lateral position. Using a scalpel and coagulation in the cutting and coagulation mode, the neoplasm was excised within the healthy tissues. After excision there was a defect measuring 20 × 17 cm (Figure 3). The skin flap was mobilized and moved to cover the defect area. The skin edges were brought together and fixed with interrupted Donati sutures using Vicryl 3-0 thread. The postoperative wound was cleaned with Iodopyrone solution and covered with an aseptic dressing.

Then the patient was transferred to a horizontal position on her back with her right arm extended to the side. Lymph node dissection was performed through a 5 cm incision in the right axillary region. Two palpable masses were identified in the dissected specimen without any evident invasion in the surrounding tissue and far from the location of the BCC (approximately 12 cm from the back tumor). The postoperative wound was treated with Iodopyrone solution and covered with an aseptic dressing.

The intraoperative material was sent for histological examination. Preoperatively, an intravenous injection of ceftriaxone in a volume of 1 g was performed. In the postoperative period, symptomatic conservative therapy with NSAIDs was carried out.

**Figure 4:**
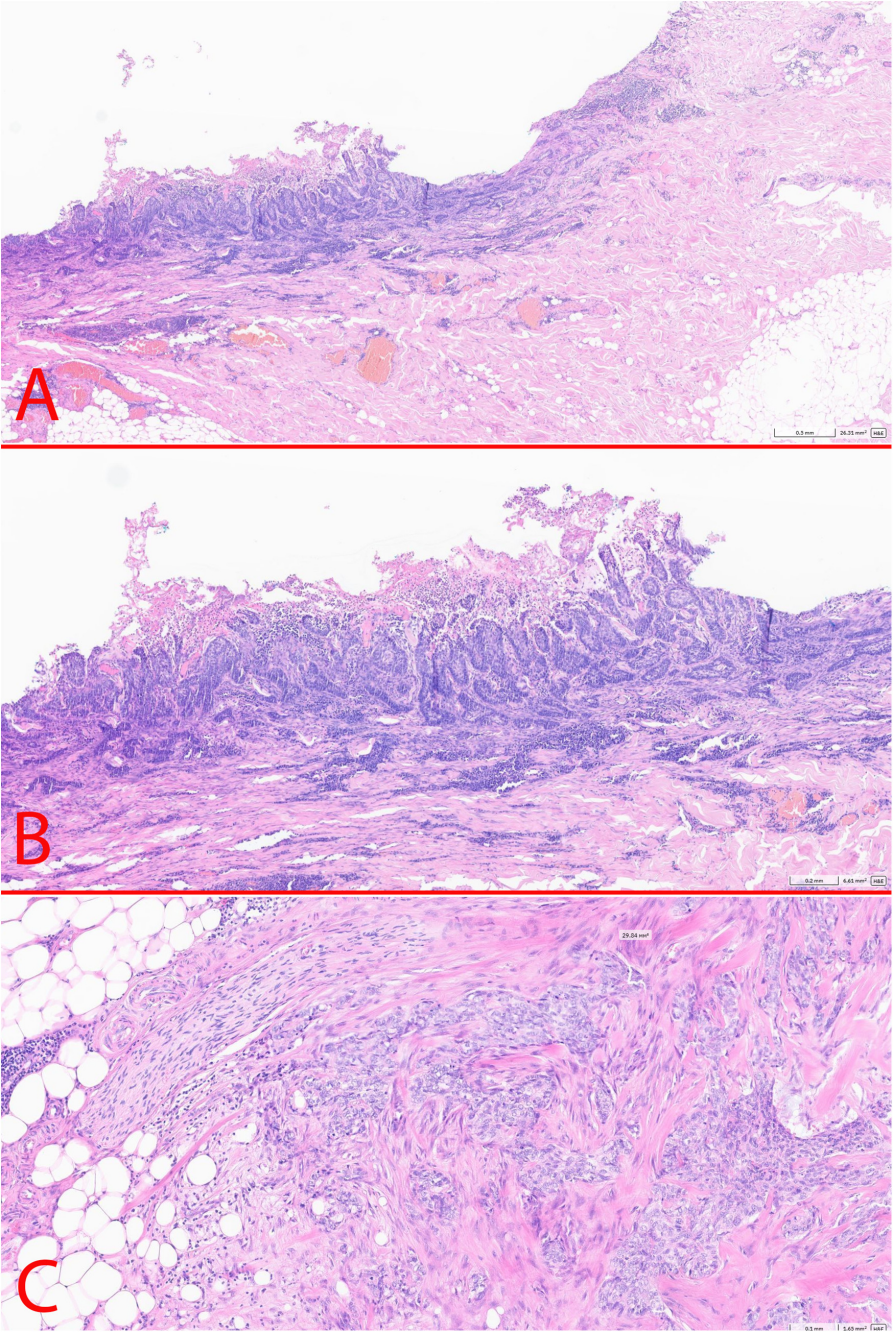
Postoperative histology. **(A)** – BCC of the skin (x2.5); **(B)** – BCC of the skin (x5); **(C)** – axillary metastasis in the subcutaneous fat (x10).

According to the histological examination of the surgical specimen (27.09.2024): the morphology corresponded to BCC (aggregates of basal cells with a small cytoplasm and large, hyperchromatic nuclei, fibromyxoid stroma) with surface ulceration, perifocal inflammation, intravascular invasion, the presence of isolated foci of tumor growth in the subcutaneous tissue (pT3 LV1 R0) (Figure 4). Two foci of BCC growth with areas of necrosis were detected in the adipose tissue. No lymph nodes were detected within the examined tissue material. Immunohistochemistry staining demonstrated GATA binding protein 3 +, cytokeratin 5/6: -, ERG: -, tumor protein P63: +, B-Cell Lymphoma 2 [Bcl-2]: +; epithelial membrane antigen [EMA]-, epithelial cell adhesion molecule BerEP4 + . Both, the back tumor and the axillary tumors had a free surgical margin (R0). As the tumors in the adipose tissue were located far from the original back lesion they were considered as metastases. Therefore, based on the histological examination the postsurgical diagnosis was BCC of the suprascapular region of the skin, T3N0M1, IV stage.

The postoperative period was uneventful. The patient underwent daily dressings of the postoperative wound and heparin ointment. Skin sutures were removed 15 days after surgery. The patient undergone a control USG 3 months after surgery with no evidence of recurrence and a chest CT scan with intravenous contrast 6 months after surgery which also did not reveal any metastasis. No recurrence of the formation was detected during the 7 months of observation of the patient.

## Discussions

There are many risk factors associated with BCC, which can be divided into external and internal. The main external factors include UV radiation (sun, tanning beds). Both intermittent and chronic insolation increase the risk of developing skin cancer. BCC can develop several years after sunburn. Long-wave UV-A1 rays emitted by the sun can penetrate the entire epidermis and reach the dermis. UV-A1 rays primarily affect basal cells, causing mutations in the p53 gene (3, 6, 7).

Internal risk factors include age, sex, Fitzpatrick phototype, and immune system status. Phototypes I, II, and III are independent risk factors for the development of BCC compared to phototypes IV–VI. There is a male predominance, however females typically are affected before the age of 40, while men over 60. BCC of the face, lower extremities, lips, and eyelids are more common in female patients, whereas BCC of the ear/external auditory canal, scalp/neck, trunk, and upper extremities were more common in males (6). Although it is the most common skin cancer, metastases have been described in only 389 cases since 1894, when Beadles first described his observation of BCC metastasizing to the lungs (4, 5). A Danish BCC study showed that metastasis from BCC occurs in 0.0001% of cases, and in patients with previously treated BCC 0.0016%–0.0083% (8). Lattes and Kessler in 1951 described 3 criteria for BCC metastasis: (1) the primary lesion and metastasis must be histologically confirmed and not predominantly squamous; (2) the primary tumor must arise from the skin and not from the salivary glands or mucous membranes; and (3) direct tumor dissemination must be excluded (9). The current basis for understanding metastatic forms of BCC is based on three literature reviews (4, 10, 11). The first review was conducted by von Domarus et al. from 1984 to 1980 and included 194 cases (10). The second review was conducted by Wysong et al. from 1981 to 2011 and included 194 patients (11). The third review was conducted by Piva de Freitas from 2011 to 2017 and included 25 cases (4). Based on these literature reviews, BCC with metastasis most often occurs in men (66%), with an average age of 45 to 65 years, with a predominant localization on the face or neck (65%) (4). Metastasis occurs in the lymph nodes (45%), lungs (35%), bones (22%), skin and subcutaneous fat (10%), liver (6%) and other localizations (4). In rare cases, metastasis occurs in the glands: parotid (1.2%), submandibular (0.7%) and thyroid (0.2%) (4).

A relationship has been described between the size of the primary tumor and the development of metastases: lesions smaller than 3 cm can metastasize in up to 2%, lesions up to 5 cm - 25% of cases and tumors larger than 10 cm have 50% chance of dissemination (12). Tumors larger than 5 cm are defined as giant and typically are associated with more tissue destruction and metastatic potential (13). The differential diagnosis includes adnexal tumors with follicular, sweat gland, or sebaceous differentiation and squamous cell carcinoma (SCC). The differential diagnoses of BCC and SCC are based on the morphologic features and immunohistochemical profiles of each individual lesion (14). SCC typically has nests of squamous epithelial cells that arise from the epidermis and extend into the dermis. These cells large with abundant eosinophilic cytoplasm and a large, often vesicular, nucleus. Compared to BCC this tumor has intraepidermal component, pagetoid spread, keratin pearls and no peripheral palisading, clefting or myxoinflammatory stroma. Immunohistochemically, the tumor is negative for BerEP4, positive for EMA, CD44, cytokeratins (14, 15). There is also basosquamous carcinoma which has a BCC and SCC area, and a transition zone between them exhibiting features of both tumors (16)

In general, the prognosis of metastatic forms of BCC is poor, and large-scale reviews have estimated the average survival at only 8–10 months (10, 11). However, these low values can be explained, at least in part, by the fact that the studies included basal squamous cell carcinoma, which is considered a variant of squamous cell carcinoma, and that they did not include patients treated with new targeted agents. Danial et al. in a study of ten patients with metastatic BCC (six of whom were treated with Hedgehog pathway inhibitors) reported much higher overall survival rates with a median of 7.3 years. The metastatic route is another factor determining survival. Longer survival was observed if the metastases were regional (median survival: 7 years and 3 months) rather than hematogenous (survival: 2 years) (17). Similarly, McCusker and coworkers demonstrated that hematogeneous dissemination yields worse prognosis than lymphatic metastases. In their study patients with distant metastases have a median survival times of 24 months compared to 87 months for regional metastasis (18).

In our clinical observation, the patient had a large tumor of 18 × 15 cm, which had been growing for 12 years. Initially, axillary lesions were interpreted as metastases to the lymph nodes, but routine histological examination revealed focal metastases to the subcutaneous fat. Treatment of BCC is usually surgical, but some forms respond to drug treatment or radiation therapy. Different types of treatment options include Mohs surgery, standard surgical excision, electrodethermocoagulation, radiation, photodynamic therapy, cryosurgery, local therapy, and systemic drugs such as Vizmodegib or Sonidegib have been proposed over the years (19). Given the size of some lesions, radical surgery may be difficult because extensive tissue defects are formed, which may require reconstruction (20).

## Conclusions

BCC is the most common skin lesion, which in isolated cases can metastasize. Metastases are mainly observed in regional lymph nodes, but hematogenous metastasis to the lungs and bones is also possible. In rare cases, metastasis to other areas occurs, as in the example of our observation in the subcutaneous fat. Considering that metastasis mainly occurs with large formations, and BCC is a slow-growing tumor, early timely diagnosis and excision of the tumor are advisable.

## Data Availability

The original contributions presented in the study are included in the article/Supplementary Material, further inquiries can be directed to the corresponding author.

## References

[1] Samarasinghe V Madan V. Nonmelanoma skin cancer. J Cutan Aesthet Surg. (2012) 5(1):3–10. 10.4103/0974-2077.94323 22557848 PMC3339125

[2] Zhang W Zeng W Jiang A He Z Shen X Dong X Global, regional and national incidence, mortality and disability-adjusted life-years of skin cancers and trend analysis from 1990 to 2019: an analysis of the Global Burden of Disease Study 2019. Cancer Med. (2021) 10(14):4905–22. 10.1002/cam4.4046 34105887 PMC8290243

[3] Gandhi SA Kampp J. Skin cancer epidemiology, detection, and management. Med Clin North Am. (2015) 99(6):1323–35. 10.1016/j.mcna.2015.06.002 26476255

[4] Piva de Freitas P Senna CG Tabai M Chone CT Altemani A. Metastatic basal cell carcinoma: a rare manifestation of a common disease. Case Rep Med. (2017) 2017:8929745. 10.1155/2017/8929745 29279714 PMC5723960

[5] Beadles CF. Rodent ulcer. Transact Pathol Soc. (1894) 45:176–81.

[6] Bernerd F Passeron T Castiel I Marionnet C. The damaging effects of long UVA (UVA1) rays: a major challenge to preserve skin health and integrity. Int J Mol Sci. (2022) 23(15):8243. 10.3390/ijms23158243 35897826 PMC9368482

[7] Wunderlich K Suppa M Gandini S Lipski J White JM Del Marmol V. Risk factors and innovations in risk assessment for melanoma, basal cell carcinoma, and squamous cell carcinoma. Cancers (Basel). (2024) 16(5):1016. 10.3390/cancers16051016 38473375 PMC10931186

[8] Nguyen-Nielsen M Wang L Pedersen L Olesen AB Hou J Mackey H The incidence of metastatic basal cell carcinoma (mBCC) in Denmark, 1997–2010. Eur J Dermatol. (2015) 25:463–8. 10.1684/ejd.2015.2546 26105129

[9] Lattes R Kessler RW. Metastasizing basal-cell epithelioma of the skin; report of two cases. Cancer. (1951) 4(4):866–78. 10.1002/1097-0142(195107)4:4<866::aid-cncr2820040424>3.0.co;2-f 14859207

[10] von Domarus H Stevens PJ. Metastatic basal cell carcinoma. Report of five cases and review of 170 cases in the literature. J Am Acad Dermatol. (1984) 10(6):1043–60. 10.1016/s0190-9622(84)80334-5 6736323

[11] Wysong A Aasi SZ Tang JY. Update on metastatic basal cell carcinoma: a summary of published cases from 1981 through 2011. JAMA Dermatol. (2013) 149(5):615–6. 10.1001/jamadermatol.2013.3064 23677097

[12] Ozgediz D Smith EB Zheng J Otero J Tabatabai ZL Corvera CU. Basal cell carcinoma does metastasize. Dermatol Online J. (2008) 14(8):5. 19061565

[13] Purnell JC Gardner JM Brown JA Shalin SC. Conventional versus giant basal cell carcinoma, a review of 57 cases: histologic differences contributing to excessive growth. Indian J Dermatol. (2018) 63(2):147–54. 10.4103/ijd.IJD_165_17 29692457 PMC5903045

[14] Cocuz IG Popelea MC Niculescu R Manea A Sabău A-H Tinca A-C Pathophysiology, histopathology, and differential diagnostics of basal cell carcinoma and cutaneous squamous cell carcinoma—an update from the pathologist’s point of view. Int. J. Mol. Sci. (2024) 25:2220. 10.3390/ijms25042220 38396897 PMC10888641

[15] Alhumaidi A. Practical immunohistochemistry of epithelial skin tumor. Indian J Dermatol Venereol Leprol. (2012) 78:698–708. 10.4103/0378-6323.102359 23075638

[16] Murgia G Denaro N Boggio F Nazzaro G Benzecry V Bortoluzzi P Basosquamous carcinoma: comprehensive clinical and histopathological aspects, novel imaging tools, and therapeutic approaches. Cells. (2023) 12(23):2737. 10.3390/cells12232737 38067165 PMC10706022

[17] Danial C Lingala B Balise R Oro AE Reddy S Colevas A Markedly improved overall survival in 10 consecutive patients with metastatic basal cell carcinoma. Br J Dermatol. (2013) 169:673–6. 10.1111/bjd.12333 23521172 PMC4006071

[18] McCusker M Basset-Seguin N Dummer R Lewis K Schadendorf D Sekulic A Metastatic basal cell carcinoma: prognosis dependent on anatomic site and spread of disease. Eur J Cancer. (2014) 50(4):774–83. 10.1016/j.ejca.2013.12.013 24412051

[19] Naik PP Desai MB. Basal cell carcinoma: a narrative review on contemporary diagnosis and management. Oncol Ther. (2022) 10:317–35. 10.1007/s40487-022-00201-8 35729457 PMC9681969

[20] Shabunin AV Lebedinsky IN Dolidze DD Bagatelia ZA Covantsev S Bocharnikov DS Giant bleeding post-traumatic thoracic sarcoma management: a case report. Front Surg. (2022) 9:1044077. 10.3389/fsurg.2022.1044077 36570811 PMC9772541

